# [Retracted] Yap regulates gastric cancer survival and migration via SIRT1/Mfn2/mitophagy

**DOI:** 10.3892/or.2024.8767

**Published:** 2024-07-01

**Authors:** Hongzhu Yan, Chengmin Qiu, Weiwei Sun, Minmin Gu, Feng Xiao, Jue Zou, Li Zhang

Oncol Rep 39: 1671-1681, 2018; DOI: 10.3892/or.2018.6252

Following the publication of the above article, a concerned reader drew to the Editor's attention that certain of the immunofluorescence data featured in Fig. 1H, TUNEL assay data in Fig. 2A, cytochome *c* leakage assay data in Fig. 2H, staining of cardiolipin images in Fig. 2H, lamellipodia-stained data in Fig. 3A, and immunofluorescence assay data in Figs. 3F and 5D were strikingly similar to data appearing in different form in other articles written by different authors at different research institutes that had either already been published elsewhere prior to the submission of this paper to *Oncology Reports*, or were under consideration for publication at around the same time (several of which have now been retracted). In addition, overlapping sections of data were noted within the data panels in Fig. 3D and F, such that data which were intended to represent the results from differently performed experiments had apparently been derived from the same original source(s).

In view of the fact that certain of these data had already apparently been published prior to the submission of this article for publication, and in view of an overall lack of confidence in the presented data, the Editor of *Oncology Reports* has decided that this paper should be retracted from the Journal. The authors were asked for an explanation to account for these concerns, but the Editorial Office did not receive a reply. The Editor apologizes to the readership for any inconvenience caused.

